# Alloreactivity of Allogeneic Mesenchymal Stem/Stromal Cells and Other Cellular Therapies: A Concise Review

**DOI:** 10.1155/2022/9589600

**Published:** 2022-03-09

**Authors:** Kiran Shah, Nirali Shah, Fatameh Ghassemi, Carolyn Ly, Teena George, Carla Lutz, Huseyin Sumer

**Affiliations:** ^1^Magellan Stem Cells P/L, Box Hill, Victoria, Australia; ^2^Department of Chemistry and Biotechnology, School of Science, Computing and Engineering Technologies, Swinburne University of Technology, Hawthorn, Victoria, Australia

## Abstract

Cellular therapies, deemed live medicine, have brought a wave of new generation biological therapies to treat previously untreatable diseases such as cancers and degenerative diseases like osteoarthritis. These cellular therapies have gained significant recognition in clinical research. The area has been further strengthened with the approval of Chimeric Antigen Receptor added on T cells (CAR-T) therapies by the regulatory authorities USA's Food and Drugs Administration (FDA), European Medical Agency (EMA), the Australian Therapeutic Goods Administration (TGA), and in many countries in 2017 to treat hematological cancers. Another milestone was achieved when allogeneic Mesenchymal Stem Cell- (MSC-) based therapy was approved by the EMA to treat Chrohn's disease in 2018. Allogeneic donor-derived MSC therapies in particular hold great promise and real hope because of their ‘off-the shelf' availability and accessibility for patients in need of urgent treatment. So far, thousands of clinical trials have explored the safety and efficacy of both autologous and allogeneic cell therapies, deeming them safe, however with varying degrees of efficacy. In the current pandemic, clinical trials have begun in many parts of the world to treat severe cases of COVID with MSCs. However, the risk of tissue rejection and the development of undesirable effects due to alloreactivity of allogeneic cells are currently not adequately addressed. Therefore, this warrants careful investigation and detailed reporting of such events by clinical researchers. This review aims at discussing the current landscape of approved allogeneic MSCs along with a few other cellular therapies. We explore any possible reactivity reported to inform the readers of any safety concern and on the efficacy of such therapies.

## 1. Introduction

**Figure 1:**
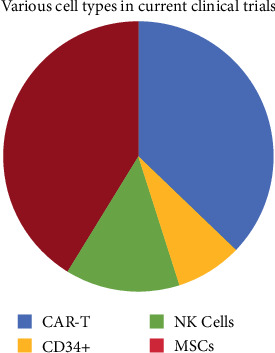
Current Clinical trials on exploring various types of stem cell or cell therapies registered on ClinicalTrials.gov.

In the current pandemic, both autologous and allogeneic MSCs have been explored in several registered clinical trials around the world to treat the severe cases of COVID-19, e.g., pneumonia and acute respiratory distress syndrome (ARDS). In such a dynamic and evolving situation dealing with a novel virus globally, a recent review describes the swift and precise targeting the COVID-19-related illness with auto/allogeneic MSC trials which demonstrates the huge therapeutic potential of MSCs in the treatment of viral infection, inflammation, and immune diseases [1]. Furthermore, the importance of stem cell therapies is highlighted by a search on the http://Clinicaltrils.gov, with the search terms: “cell therapy”, “stem cells”, “active” and “recruiting by invitation”, and “interventional” resulted in 9,949 phase I to phase III studies currently registered. These studies are investigating 25 conditions under category for over recruiting (http://Clininicaltrials.gov, accessed 08 Feb2021; https://clinicaltrials.gov/ct2/results?term = cell+therapy+OR + stem+cells&recrs = a&recrs = f&recrs = d&age_v = &gndr = &type = Intr&rslt = &Search = Apply). There are over 530 clinical trials focusing on the allogeneic stem cell therapy and have several different types of stem cells being used, e.g., hematopoietic stem cells (HSCs: CD34/NK), MSCs, and CAR-T cells and 629 clinical trials with autologous cell sources.  Figure 1 shows the distribution of the cell types currently listed on ClinicalTrials.gov.

In recent times, cell therapies have had unprecedented progress, exploring a wide range of adult stem cells other than hematopoietic stem cell transplantation (HSCT) for treatment of a number of disease through the use of MSCs, Natural Killer Cells (NK Cells), skin cells, T cells, and more. With this enormous progress, a milestone was achieved with the approval of one of the first cell therapies to treat Graft Versus Host Disease (GvHD) in Canada and New Zealand in 2015 followed by EMA and a recent approval of CAR-T therapy, Kymriah by FDA in 2018 [2, 3]. The regulatory approvals highlight that these cellular therapies hold great promises to address current unmet medical needs including potential treatment and cure of several deadly and life-threatening diseases. Consequently, cellular therapies have gained significant clinical advances with several thousands of clinical trials registered in the ClinicalTrials.gov database. These include both autologous and allogeneic cell therapies treatment modalities, each with its own set of advantages and disadvantages. Due to the multipotent therapeutic properties of MSCs, in the current pandemic, over a dozen clinical trials are underway for the treatment of COVID-19. This review will explore the current cellular types in the clinical trials with special focus on the most common cell types. We discuss the risks and benefits of autologous versus allogeneic and list the current approved cell therapy available to patients.

Over five decades ago, a ground-breaking bone marrow transplant in a sibling with a close histocompatible match was performed and heralded a new era of treating complex medical conditions. Thus, a brand-new field of therapy was uncovered and termed stem cell therapy. To date, allogeneic bone marrow transplantation (BMT) is a treatment option for a range of hematologic diseases including acute myeloid leukaemia. HSCT derived from donor bone marrow has been an established therapeutic method for over 5 decades. The curative action of HSCT is due to immune-mediated graft-versus-leukaemia effects of the donor T cells contained in the graft. However, donor T cells are also the cause of Graft-vs-Host disease (GVHD). As GVHD is a major complication arising from allogeneic HSCT, research into the prevention of GVHD has provided a unique opportunity to understand the mechanisms of alloreactivity. One such mechanism that causes severe GVHD is the production of antirecipient helper (Inerleukin-2-producing) T lymphocyte precursors (HTLp). These precursors amplify the immune response as shown by a positive correlation between patients with high HTLp and the development of GVHD [4]. These findings are supported by an earlier study where depletion of T cells from donor bone-marrow allografts prior to transplantation prevented the development of GVHD [5].

## 2. Autologous versus Allogeneic Stem Cell Therapies

There are number of advantages of using autologous (self-derived) cellular treatment including the following: (i) the autologous cell therapy poses a low risk of tissue rejection, (ii) less chance of getting foreign infections, and (iii) lower risk of acquiring an unwanted immune response. However, this modality is costly, time consuming, and in some cases may not be efficacious, especially in chronically ill patients with potentially compromised cell quality or inability to donate tissue due to potential surgery risk. Furthermore, the self-derived tissue may also contain underlying genetic factors that are the cause of the disease. Allogeneic (donor-derived) cell therapy also has some issues including (i) the suitability for a diverse population, (ii) chance of carrying foreign antigens that could illicit unwanted immune response, and (iii) tissue rejection. However, if these limitations are overcome with careful health screening of donors; then, allogeneic cell therapy presents many added advantages over the autologous therapy. These include the following: it is less expensive, ‘off the shelf' and therefore immediately available, and well-characterised allogeneic donor cell lines with potentially increased efficacy. However, the issue with developing allogeneic reactivity in the long term remains the main concern for the allogeneic cell therapy and warrants better long-term follow-up and reporting in clinical trials.

In this review, given the perceived risks discussed above with allogeneic antigens in the recipients and possible tissue rejection, we reviewed the scientific literature for evidence of alloreactivity and rejection and summarise the current status of alloreactivity and allogenic MSC cell therapies.

## 3. Allogeneic Stem Cell Therapy and the Role of Major Histocompatibility Complex (MHC)

The MHC group of genes determines and regulates the unique molecular constituents of specific antigens and their interaction with the various immune cells. Therefore, the molecular recognition of foreign cells originates at the genetic level and the peptides on the MHC molecules contribute to allorecognition. Of the three types of MHC gene family subgroups, classes I and II are directly involved in the antigen presentation process. Class MHC class III are structurally and functionally less well defined and the gene cluster contains signalling molecules such as TNF-alpha and heat shock protein. Classes I and II therefore orchestrate the allogeneic reactivity in the recipients' immune cells [6]. In cellular transplantations, the recipient's T cells act as major coordinator for specific immunity, facilitated by the antigen-binding site of class I and II molecules, targeting allogeneic donor's epitopes or the MHC complexes. The molecular basis for this interaction, termed alloreactivity, occurs in three different ways; first, the heterogeneous structural differences in the stimulator and the responder MHC types in allorecognition; second, alloreactive T cells may not be adequately recognised by the responder cells when closely related; and third, alloresponse may be directed against the residues on the allogeneic MHC molecules itself [6].

Many other immune cells, e.g., macrophages, B-cells, and Natural Killer (NK) cells are primed to illicit strong reactive immune responses when nonself cells or tissues are introduced. This can then trigger undesirable tissue rejection and cause severe morbidity in the recipient. These cells express high levels of class I and II MHC complexes. For this reason, several preventative measures are employed before allogeneic cell or tissue transplantation. One of many measures is cytotoxic cross-match, and together with Allomap Molecular Expression testing and ABO-compatibility, to help predict the success of transplantation [6]. With modern advances in cellular biology, many innovative and noble approaches are employed in various clinical trials to develop successful allogeneic cell therapies such as CAR-T cell therapy, CRISPR- and iPSC-derived cells, and other naturally immune-evading stem cells such as MSCs. We explore the alloreactivity of these different cell types in the sections below.

## 4. Types of Allogenic Stem Cell Therapies in Clinical Trials

### 4.1. Mesenchymal Stem Cells (MSCs)

MSCs are one of the most studied therapeutic cell types; they are adult stem cells and derived from a number of connective tissues such as the bone marrow, adipose tissue, umbilical cord, placenta, and dental pulp. With the regulatory approval of the use of allogeneic MSCs for acute GvHD, the field has gained a renewed confidence in the therapeutical benefits of these multipotent cells [7]. These cells are widely known as being immune-privileged due to the lack of MHC II molecule expression and a very low level of MHC I [8, 9]. Additionally, potent stimulatory molecules such as CD86, CD40, or CD40L are not detectable in MSCs and therefore not recognised by alloreactive T cells. As a result these cells do not get rejected by the host [9]. Importantly, MSCs are now widely described as strong immunomodulators that have been shown to have antiproliferative effects on several immune cells including T cells, B-cells, and NK cells [10–12]. The underlying mechanisms of T cell inhibition by MSCs are facilitated by several key molecules including the enzymes Indoleamine 2,3 dioxygenase (IDO), Prostaglandin E2 (PGE2), Human Leukocyte Antigen -G (HLA-G), Nitric Oxide, Insulin Growth factors (IGF) binding proteins, and secreted exosome cargo [8, 13]. Depletion of tryptophan by an enzyme IDO produced by MSCs seems to have immune suppressive effects on the T cells [14]. Another molecule responsible for immune suppression is PGE2 which has been shown to convert preinflammatory T helper cells that secrete interferon gamma into T helper cells to produce anti-inflammatory cytokines IL-4 [15].

Le Blanc and coworkers first used allogeneic MSCs clinically to treat acute GVHD [16]. This landmark case study inspired researchers to apply allogeneic MSC in different clinical trials. Based on systematic analysis of MSCs clinical trials that Kabat and coworkers [17] have published last year, there is a dramatic increase in the number of newly registered clinical trials using MSCs as most recently reported in April in 2021 [1]. An outcome result of allogeneic canine MSCs treatment for lame dogs showed good efficacy and without any adverse reaction of the allogenic MSCs [18]. There are a few animal studies which have shown adverse response upon administration of allogeneic MSCs [19–21]; however, allorejection-dependent symptoms have not been reported in humans [22]. The safety of allogeneic MSC therapy and lack of serious adverse effects or toxicity have been consistently demonstrated in clinical trials, despite this, the secretion of alloantibodies has been reported in some studies [23, 24]. Furthermore, efficacy of allogeneic therapies is correlated with different factors such as cell dose, dose frequency, administration route, and source of MSC. The first and most widely used source of MSCs is derived from the bone marrow. Allogeneic MSC recipients did not display significant donor-specific antibodies with only 3.7% alloreactivity in the allogeneic group [25], while MSC therapy in both allogeneic- and autologous cell-treated groups were safe. However, in this study there was limited clinical improvement in both groups [25]. Although no serious adverse effects were seen after one month, long-term adverse effect incidence after 6 month and rehospitalization after one year in 20 million cell dose group were 2-fold higher than that of 100 million dose group. The authors attribute this anomaly to the complex disease environment and suggest that an even higher dose may be required to overcome the initial cell death and hypoxia after transplantation [25]. The study demonstrated no clinical alloreactivity in patients and both doses were considered safe and well-tolerated, while only the higher dose displayed efficacy on reduction of infarct size and improving cardiac function [25]. Similarly, in a study where a dose comparison was made of allogenic mesenchymal precursor cells (MPC) in patients with heart failure, it was demonstrated that the higher dose (150 million cells per patient) produced the greatest improvement in cardiac structure and function [23]. Results also showed that 11% of the MPC-treated patients developed donor-specific anti-HLA class I antibodies but no serious adverse events were attributed to MPC treatment [23]. Mismatched patients in the treatment groups may have been a factor as well as the requirement to administer a high cell dose [26]. To overcome these obstacles, encapsulation of allogeneic MSCs before their transplantation has been proposed which prolongs their survival and subsequently ensures long-term efficacy of the allogeneic cell therapies [27]. Activating MSCs with proinflammatory cytokines such as interferon gamma (IFN-g) and/or tissue necrosis factor-alpha (TNF-a) has also been recommended by the ISCT MSC committee to further enhance the effectiveness of these cells [20]. Allogeneic cell therapies also have added advantages over autologous cells due to the limitations of obtaining adequate tissue from already diseased patients with chronic conditions and possibly compromised efficacy in cells from such tissues. Currently, there are over a dozen clinical studies aimed at developing better delivery systems in various cell therapies such as heart conditions or degenerative disc disease using injectable hydrogels in combination with MSCs providing a better treatment model [28–30]. With a wide array of untreatable conditions being targeted by these promising off-the-shelf MSC therapies, continued and careful long-term monitoring of the implanted cells in the recipients still needs to be carried out even though allogenic MSC therapies have been shown to be safe. In particular, we would like to draw attention to new cohorts of patients with new illnesses being treated with MSCs to have close diligent clinical follow-up to assure patients of continued safety and efficacy.

### 4.2. CAR-T Cell Therapy

**Figure 2:**
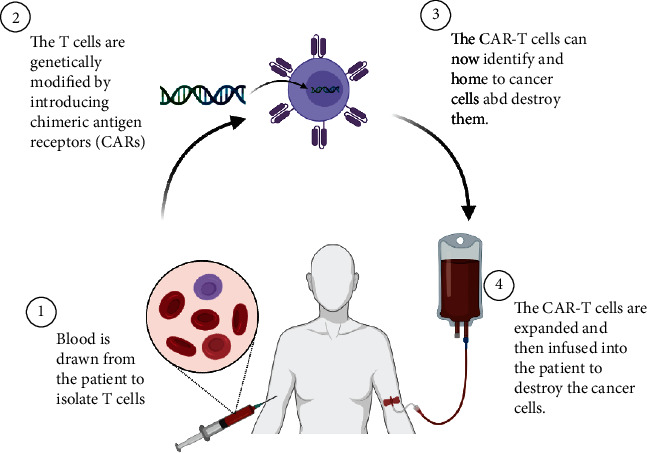
CAR-T cell therapy overview. The patients' T cells are isolated and genetically modified with a chimeric antigen receptors (CARs) that can home to cancer cells when infused back into the patients' blood stream.

CAR-T cell therapy is one the most novel immunotherapies of the 21st century, it is used for cancer treatment and has been explored in many clinical trials globally. The process of Chimeric Antigen Receptor (CAR) is an engineered T cell receptor with the ability to recognise a predefined target antigen. It is genetically introduce to the patients' T cells which helps to activate cytotoxicity against the target cells, i.e., malignant cells. It has been described as an antitumour weapon, which acts directly in the immune system (Figure 2). A milestone was achieved recently when Kymriah and Yescarta received approval from the regulatory authorities FDA, EMA, and TGA to use CAR-T cell for commercial therapies in eligible patients [2, 3].

Furthermore, Adoptive Cell Transfer (ACT) therapy is an immunotherapy that separates immunocompetent cells in cancer patients and transfers them back in the patients after expansion or functional identification in vitro. The adoptive cells kill tumour cells directly or stimulate the body's own immune response. There are ‘four-generations' of CAR-T cells that have been used in ACT therapy [31]. The first generation includes Single Chain Fragment Variable (ScFV) as the target recognition and CD34 signalling chain as the intracellular domain. The second generation encompasses a costimulatory domain such as 4-1BB (CD137) or CD28 as the secondary signal producer, in addition to properties of the first generation. Applying both costimulatory domain including CD28 and 4-1BB led to the construction of the 3rd generation. The fourth generation, also named as T cells Redirected for antigen-Unrestricted Cytokine-initiated Killing (TRUCK T cells) or armed CAR-T cells. To increase efficiency and potency, various functional elements such as interleukin genes are inserted into fourth generation CAR construction [32].

As discussed above, autologous therapies are more expensive and time-consuming, allogeneic donor-derived CAR-T provides an advantage over the autologous approach. Healthy donors with distinct HLA types generate ‘off-the-shelf' CAR-T cell banks that can and be matched with the recipient HLA alleles for precision medicine and therefore avoid the risk or incidence of allograft rejection [33]. In our observations of the registered 829 clinical trials currently registered for of CAR-T cells on Clinical Trials.gov, 53 of these have completed phase I/II trials and 13 of these completed trials have results published on the website with mixed results. These early results warrant further follow-up and continued monitoring of the long-term safety of these procedures. While the data from the remaining ongoing CAR-T cell trials will be of great interest to immunotherapy researchers and cancer patients alike.

### 4.3. CD34 Positive Cells

**Figure 3:**
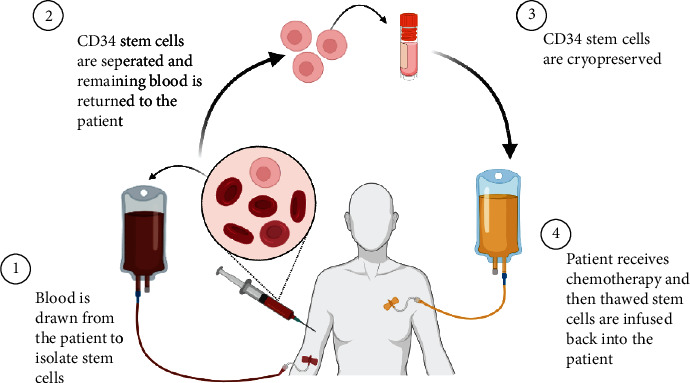
CD34 cell therapy overview. The patients' blood is drawn and CD34 positive cells are isolated and expanded in culture before cryopreservation. The CD34 cells can be infused back into the patient after they receive chemotherapy.

The ability to select CD34+ cells from mobilized peripheral blood makes it feasible to expand these purified cells in cytokine-supported cultures for clinical use. The transplantation of allogeneic Peripheral Blood Progenitor Cells (PBPC) provides complete and sustained hematopoietic and lymphopoietic engraftment (Figure 3). The positive selection of CD34+ cells from peripheral blood preparations simultaneously provides an approximately 1000-fold reduction of T cells. These purified CD34+ cells contain committed multipotent stem cells that are suitable for allogeneic transplantation [34]. Both CD34+ endothelial and cardiomyocyte progenitor cells are capable of neoangiogenesis/neovascularization and cardiac muscle regeneration, respectively, and can be easily collected in humans from the bone marrow or the peripheral blood. The experimental and physiological data support the use of CD34+ stem cells for cardiac repair [35]. For example, patients with severe diffuse coronary artery disease (CAD) receiving intracoronary transfusion of circulating-derived CD34+ cells directly without enrichment for endothelial progenitor cells (EPCs) were divided into angiographic low- and high-score groups after a 9-month follow-up [36]. The results of cardiac MRI and 3D echocardiography demonstrated a significant improvement of the left ventricular ejection fraction (LVEF) in the high-score subjects compared to that in their low-score counterparts. Moreover, the neovascular effects were demonstrated in a phase I/II trial, where CD34+ EPCs administered to patients with refractory angina pectoris decreased the frequency of events and increased exercise tolerance as compared to the placebo-treated patients [37]. The donor's alloreactive effector T cells and the host's antigen-presenting cells (APCs), such as dendritic cells, have been shown to have an essential role in the initiation of GVHD. Depletion of effector T cells from the HSCT graft decreases the risk of GVHD but at the same time increases the relapse rate, due to insufficient graft-versus-leukaemia (GVL) effect [38]. Therefore, these treatments further warrant careful review and fine-tuning of the treatment regime, dosages, modalities, and long-term safety evaluation.

### 4.4. Skin Stem Cells

**Figure 4:**
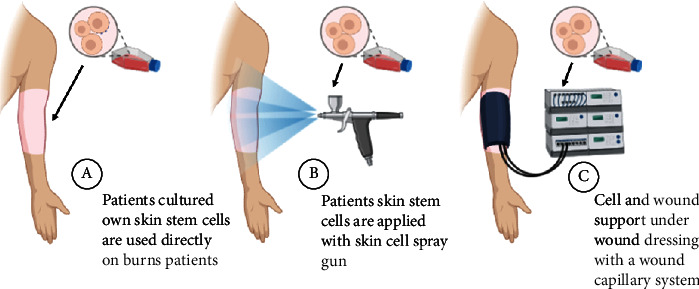
Skin cell therapy overview. Patients own cultured cells or allogeneic cells can be (a) used directly on the wound site, (b) applied using as an aerosol, or (c) cell replacement supported under a wound dressing with a wound capillary system.

Cell therapy has been a breakthrough for major burn victims for permanent skin replacement using cultured autologous epidermal grafts (Figure 4). Rheinwald and Green first established the culture of keratinocytes in vitro in 1975 [39]. In the following years, further research led to the first autologous keratinocyte cell treatment for a burn patient in 1981 [40]. Allogenic keratinocyte cell therapy followed in 1983, where wound healing was observed at an unexpectedly quicker rate compared to the conventional procedure of using cadaver autografts [41]. However, as one of the limitations with allogenic cell therapy is the potential for immunological rejection from the recipients' immune system, whether these MHC class II antigens on keratinocytes can initiate an immunological response is still largely debated with some studies showing no detectable expression of these antigens on the cell surface [42], while other studies have been able to show expression of HLA-DR on keratinocytes after IFN-*γ* exposure [43, 44].

Keratinocytes, like all epidermal cells, have MHC class I antigens displayed on their cell surface but the human leukocyte antigen type DR (HLA-DR), a MHC class II antigen strongly associated with immune rejection, is not constitutively expressed [44]. In an early cell therapy experiment by Morhenn et al. in 1982 examining the levels of HLA-DR expression, keratinocyte cells were stained with an anti-HLA-DR antibody [42]. This examination resulted in no detectable fluorescence even after the cells were left in culture for up to 14 days. This team also discovered significantly reduced lymphocyte activation levels in a coculture experiment of keratinocytes and lymphocytes after a seven-day period, compared with freshly harvested keratinocytes. These results suggest that the antigenicity of keratinocyte cells in culture is reduced over time.

However, other studies have shown that these MHC class II antigens on keratinocytes can be activated by stimulation with the proinflammatory cytokine IFN-*γ*. Castillo et al. examined the immunogenicity of cultured allogenic keratinocytes by coincubating them with peripheral blood mononuclear cells and IFN-*γ* [45]. They found that despite MHC class II molecules being activated by up to six times greater than baseline levels, T lymphocyte activation markers were not detected in this assay [43, 45]. Similar results were also observed where IFN-*γ* was able to induce HLA-DR activation in keratinocytes [44]. However, key T cell activation costimulatory molecules B7-1 and B7-2 could not be detected, even with high levels of IFN-*γ* exposure in the coculture experiment. These results suggest that HLA-DR is not actively expressed on keratinocytes and these cells have been described as being nonprofessional antigen presenting cells with their limited immunogenicity [44].

## 5. Approved Allogenic Cell Therapy Treatments

With extensive clinical research in cellular therapies, there currently is a number of products that have been approved by various regulatory authorities. Currently the FDA has 19 cell therapy products approved for use in patients (https://www.fda.gov/vaccines-blood-biologics/cellular-gene-therapy-products/approved-cellular-and-gene-therapy-products).The first cell therapy product to get regulatory approval for therapeutic use was in August of 2017 for relapsed or refractory (r/r) paediatric and young-adult B-cell acute lymphoblastic leukaemia and then in May 2018 for adult r/r diffuse large B-cell lymphoma [46]. The CAR-T cell therapy trial outcome data showed significant improvement and overall response rate of 50% and in some cases it was >80% [47]. CAR-T cell therapies now represent the new standard of care for select groups of patients with B-cell lymphoma and have now been approved by the FDA, EMA, and the TGA [2].

Half a century since the discovery of the multipotency and regenerative properties of MSCs and thousands of clinical trials exploring their therapeutic benefits for several dozens of clinical indications, there are a few success stories that are emerging with successful clinical outcomes and regulatory approvals. MSC cell therapy has gained regulatory approval to address the unmet clinical needs in the areas of graft-versus-host disease, Crohn's disease, and critical limb ischemia [48]. Recently, adipose-derived MSCs were approved for the treatment of Chrohn's disease by EMA in 2018, called Alofisel [49]. The results published from this pivotal study of over 200 patients showed 56% of patients in the trial achieved lifesaving remission at 52 week posttreatment [49].

Table 1 adapted from Levy, et al. (2020) [50].

Asia is leading the way in approving and supporting these breakthrough cellular and gene therapies that have been termed Advanced Therapy Medicinal Products (ATMPs) [50]. In a 2019 report, EMA had approved fourteen ATMPS for use to address the unmet clinical need in patients and for some approved ATMPS. Some countries also support these breakthroughs but expensive therapies through subsidies. Furthermore, there is debate throughout Europe to financially support the development and subsidise the use of these therapies in order to increase accessibility to patients in need [51, 52].

## 6. Conclusion

Allogeneic cell therapies are “off-the-shelf” options that provide innovative, efficacious, and safe treatment solutions for many of the untreatable diseases. Allogenic cell therapies are currently in use in many clinical trials and have also been approved for clinical use in several jurisdictions. Our review provides evidence that the alloreactivity risks of the implanted allogenic cells to the recipients are minimal and do not cause severe adverse reactions in patients. With several approved cell therapies, this heralds a new era of cell therapies for previously untreatable diseases such as Chrohn's disease and GvHD. However, many new cellular therapies that are in various stages of clinical trials and currently underway for the treatment of cancers and other degenerative medicine need close monitoring and stringent reporting of any unexpected adverse reactions along with the efficacy data. Cell therapy is an exciting emerging field and the continued reporting of the clinical outcome data is vital to help strengthen the future of this very promising modern live medicine.

## Figures and Tables

**Table 1:** Current MSC-approved products for human clinical use.

Source of human tissue	Clinical condition	Trade name	Company	Approving country and year of approval
Adipose	Complex perianal fistulas in CD	ALOFISEL	TiGenix NV/Takeda	Europe (2018)
Bone Marrow	Spinal cord injury	STEMIRAC	Nipro Corp.	Japan (2018)
Bone Marrow	Critical limb ischemia	STEMPEUCEL	Stempeutics Research PVT	India (2016)
Bone Marrow	GvHD	TEMCELL HS INJ	JCR Pharmaceuticals	Japan (2015)
Bone Marrow	Amytrophic lateral sclerosis	NEURONATA-R	Corestem Inc.	South Korea (2014)
Bone Marrow	GvHD	PROCHYMAL	Osiris Therapeutics Inc./	Canada (2012)
		(REMESTEMCEL-L)	Mesoblast Ltd.	New Zealand (2012)
Adipose	Crohn's fistula	CUPISTEM	Anterogen Co. Ltd	South Korea (2012)
Umbilical Cord	Knee articular cartilage defects	CARTISTEM	Medipost Co. Ltd.	South Korea (2012)
Bone Marrow	Acute MI	CELLGRAM-AMI	Pharmicell Co. Ltd.	South Korea (2011)
Adipose	Subcutaneous tissue defects	QUEENCELL	Anterogen Co. Ltd.	South Korea (2010)
